# Fixed Dystonia of a Limb: Amputation Could Be a Favourable Outcome

**DOI:** 10.7759/cureus.94671

**Published:** 2025-10-15

**Authors:** Rajdeepsinh H Rana, Shivani Virpura, Yogendrasinh Jagatsinh

**Affiliations:** 1 General Internal Medicine, Cumberland Infirmary, Carlisle, GBR; 2 Obstetrics and Gynaecology, Cumberland Infirmary, Carlisle, GBR; 3 Stroke and Rehabilitation Medicine, Cumberland Infirmary, Carlisle, GBR

**Keywords:** amputation, fdl: fixed dystonia of limb, high arched foot deformity, microprocessor-controlled prosthetic knee, phantom limb pain

## Abstract

Fixed dystonia of limb is a rare and complex disorder characterized by abnormal, fixed posturing of a limb, often accompanied by chronic pain. Its etiology remains controversial, with debates surrounding psychogenic versus organic origins. We present the case of a 22-year-old female with a longstanding history of left foot deformity and progressively worsening neuropathic pain, which persisted despite multiple pharmacological treatments, physiotherapy, and psychological interventions. The patient developed severe psychological distress, including suicidal ideation and a self-amputation attempt. After thorough multidisciplinary evaluation, an above-knee amputation was performed. Postoperatively, she experienced complete resolution of preoperative pain, minimal phantom pain managed conservatively, and regained full independence with a prosthesis. Her functional capacity and mental health improved significantly. This case highlights the extreme challenges of managing fixed dystonia and suggests that amputation, although controversial, may be beneficial in highly selected patients with intractable symptoms and significant psychosocial impact.

## Introduction

Dystonia refers to a group of movement disorders characterized by sustained or intermittent muscle contractions leading to abnormal, often repetitive movements or postures [1]. Fixed dystonia of limb (FDL) is a rare subtype in which posturing becomes persistent and resistant to voluntary correction. The condition often follows minor peripheral trauma or injury and predominantly affects young women [1,2]. While exact prevalence is unknown, FDL is estimated to account for a small fraction of all dystonia cases and has been described in fewer than a few hundred documented patients worldwide [1,3].

The etiology of FDL remains controversial. Historically, it was viewed as a psychogenic disorder, often associated with psychological distress or conversion phenomena [1,4]. However, recent neurophysiological and neuroimaging studies suggest functional abnormalities in central nervous system processing - such as altered sensory temporal discrimination and disrupted body representation - without any structural brain lesions [1,3,4]. This supports the classification of FDL within the spectrum of functional movement disorders (FMDs) rather than purely psychogenic conditions. Nevertheless, the overlap between psychological factors, functional reorganization, and maladaptive sensorimotor control contributes to its complexity [3,4].

Clinically, patients present with sustained abnormal limb postures that may be painful and are often triggered or worsened by minor injuries. Symptoms can evolve from intermittent spasms to fixed posturing, leading to muscle contractures and disability [1,3]. In many cases, pain radiates beyond the dystonic limb and may exhibit neuropathic features. Psychiatric comorbidities, including anxiety, depression, or post-traumatic stress symptoms, are frequently reported [1,2,4].

The pathophysiology involves altered sensorimotor integration, impaired proprioceptive feedback, and abnormal cortical excitability [1,3,4]. Studies demonstrate that patients with FDL show abnormal activation patterns in the supplementary motor area and basal ganglia during movement tasks, supporting central nervous system dysfunction as a key mechanism [3,4].

FDL is distinct from complex regional pain syndrome (CRPS), although overlap may occur. CRPS typically follows trauma and features vasomotor, sudomotor, and trophic changes with pronounced swelling and temperature asymmetry [5]. In contrast, FDL involves sustained posturing without autonomic or trophic manifestations, and imaging or neurophysiological studies show no structural lesion or ongoing nerve damage [1,5].

Management of FDL is multidisciplinary. Pharmacologic therapy includes muscle relaxants, botulinum toxin injections, neuropathic pain agents, and antidepressants [1,4]. Physiotherapy and psychological interventions - particularly cognitive-behavioral therapy and graded motor imagery - are essential to improving mobility and reducing disability. Despite these approaches, outcomes remain variable, and many patients continue to experience severe pain and fixed postures.

In rare, treatment-refractory cases, amputation has been considered as a last resort [2,6,7]. However, this approach is ethically complex and clinically controversial due to the risks of persistent pain or recurrence in the residual limb [7]. Nonetheless, in highly selected cases where quality of life is severely impaired, amputation may lead to substantial physical and psychological improvement [2,6,7].

Given the rarity and controversy surrounding this intervention, this case report aims to illustrate the natural history, management challenges, and postoperative recovery of a young woman with severe, therapy-resistant FDL who achieved remarkable functional and psychological improvement following above-knee amputation.

This case was previously presented as a poster at the British Society of Physical and Rehabilitation Medicine Annual Conference on 08/11/2024.

## Case presentation

A 22-year-old female with a history of high-arched foot deformity (2015) developed progressive inversion of the left foot. Patient described her pain as a constant stabbing sensation radiating from her back down the left leg and foot, with intensity fluctuating between 6/10 on her best days and 9/10 at its worst, averaging 8/10 at the time of clinical evaluation (2019). The pain was aggravated by lying down, prolonged sitting, and walking, though this improved somewhat after transitioning to a hospital bed. On examination, associated features included allodynia, hyperalgesia, and paresthesia. A DN4 questionnaire yielded a score of 6/10, supporting a neuropathic profile. Mechanical causes were excluded through imaging, including MRI of the spine and lower limb, which showed no evidence of structural abnormalities or nerve compression. Inflammatory etiologies were considered but excluded on the basis of normal inflammatory markers and absence of joint swelling or warmth. Electrophysiological studies (nerve conduction and EMG) demonstrated no evidence of radiculopathy or entrapment neuropathy. Taken together, these findings supported a diagnosis of fixed dystonia with neuropathic pain rather than mechanical or inflammatory pathology.

Clinical examination

On physical examination, the patient’s left lower limb demonstrated a fixed dystonic posture. The foot was persistently inverted with internal rotation at the ankle, and the patella was oriented medially and slightly downward in the sitting position, consistent with proximal spread of dystonia. Passive correction of the deformity was not possible. Muscle tone was markedly increased, with uniform resistance to passive movement at both the ankle and knee, though no spastic catch was observed. Sensory testing revealed allodynia, with pain elicited by light touch, and hyperalgesia in response to pinprick stimulation, particularly over the dorsum of the foot, where the patient also reported intermittent paresthesia. Despite these sensory abnormalities, motor strength was preserved throughout both proximal and distal muscle groups, and no focal weakness was detected. Deep tendon reflexes were symmetrical and within normal limits, and examination of other systems yielded no abnormal findings.

Functional evaluation and imaging studies

Gait assessment revealed a markedly antalgic pattern characterized by persistent inversion and internal rotation of the left foot, accompanied by restricted knee flexion and compensatory abduction at the hip during ambulation. The patient was able to walk short distances using handheld support but required a wheelchair for community mobility. The range of motion at the ankle and subtalar joints was completely restricted, and no passive correction could be achieved. Knee movement was limited by dystonic muscle activity involving the quadriceps and hamstrings, and the patella was observed to rotate medially and slightly downward in the sitting position.

Magnetic resonance imaging (MRI) and plain radiographs of the pelvis, hips, and knees demonstrated normal joint morphology without evidence of arthritis, effusion, or structural abnormalities. There were no periarticular soft tissue signal changes, effectively excluding mechanical, inflammatory, or neurogenic causes.

Pain assessment using the Visual Analog Scale (VAS) ranged from 6 out of 10 on the best days to 9 out of 10 on the worst days, and the DN4 questionnaire yielded a score of 6 out of 10, confirming the presence of neuropathic pain features. Mobility assessment showed significant functional limitation, as the patient required assistance for walking and frequently used a wheelchair for longer distances. Psychologically, the Hospital Anxiety and Depression Scale (HADS) revealed elevated scores for both anxiety and depression, reflecting severe psychosocial distress and reduced quality of life.

Over a four-year period, the pain progressively worsened, resulting in marked loss of independence and social withdrawal. Multiple conservative management strategies were attempted, including intensive physiotherapy, transcutaneous electrical nerve stimulation (TENS), and pharmacologic interventions such as paracetamol, ibuprofen, duloxetine, pregabalin, and combined paracetamol with codeine phosphate. Despite these efforts, symptomatic relief remained minimal. The patient underwent evaluation by pediatricians, chronic pain specialists, psychologists, and orthopedic surgeons, none of whom identified a mechanical or inflammatory etiology to account for her fixed dystonic posturing.

**Figure 1:**
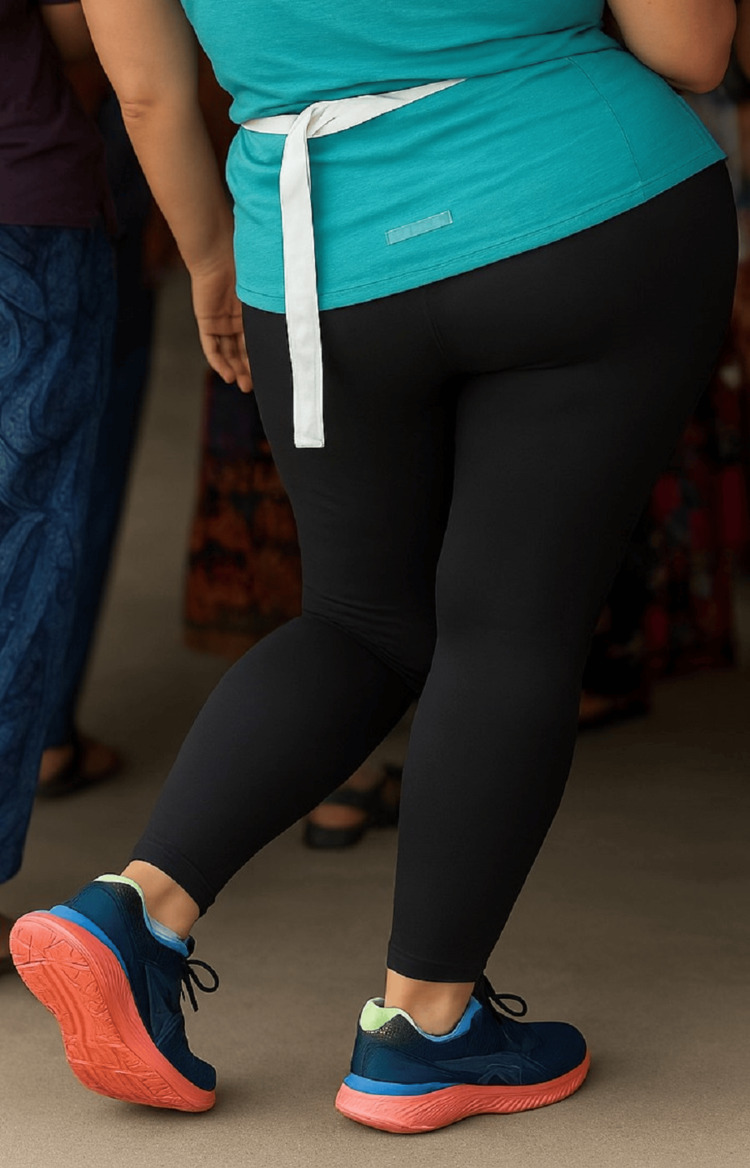
Positioning of the left limb while standing Left lower limb at initial presentation (2018), demonstrating fixed inversion of the foot.

**Figure 2:**
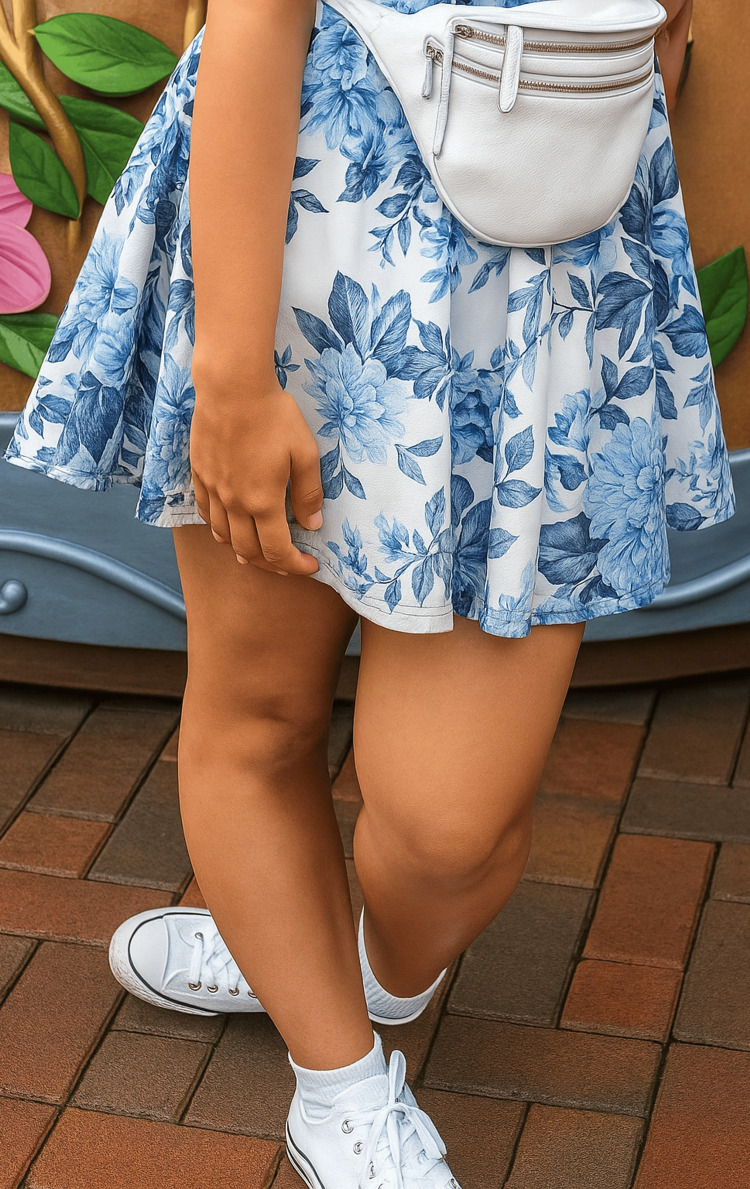
Positioning of the left limb while standing Same limb two years later (2020), showing progression of dystonia with marked inversion, internal rotation.

Figures 1, 2 show the abnormal positioning of the patient’s left limb while standing, highlighting the fixed inward rotation and rigid posture.

Social stressors, including bullying at school and threats of violence, contributed to her social withdrawal, poor sleep, and worsening mental health. She expressed that her quality of life might improve if her leg were amputated. In 2020, she attempted self-amputation using a saw, leading to severe depression and self-harm behaviors. 

**Figure 3:**
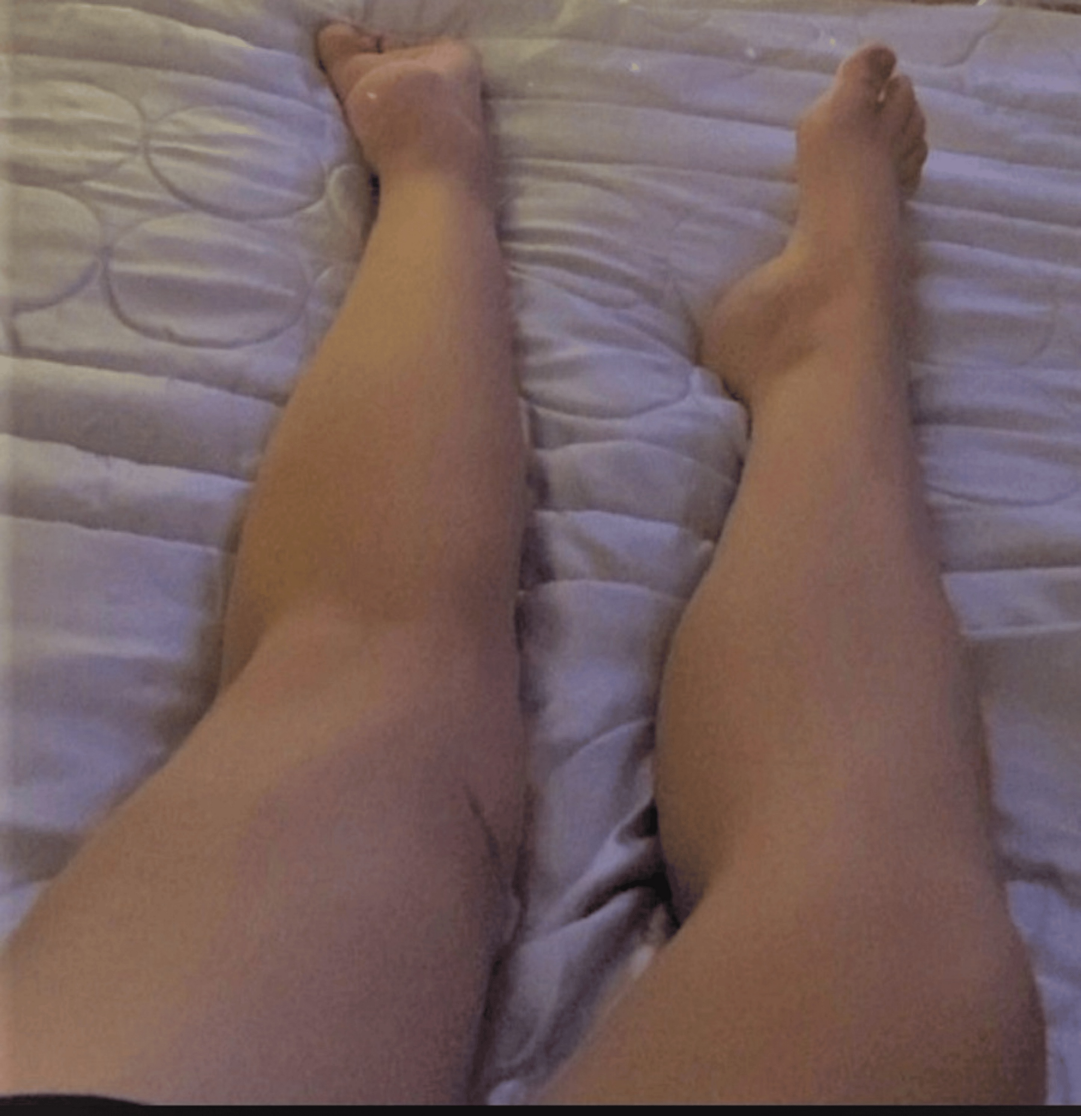
Position of the left limb in bed in supine position Clinical photograph taken six months after Figure 2, demonstrating further progression of the deformity with marked inversion and internal rotation. The patient is lying supine but not in strict dorsal decubitus, as the right limb is also slightly rotated to the right side.

The patient’s posture while lying supine is depicted in Figure 3, further emphasizing the fixed dystonic configuration of the limb in various positions.

**Figure 4:**
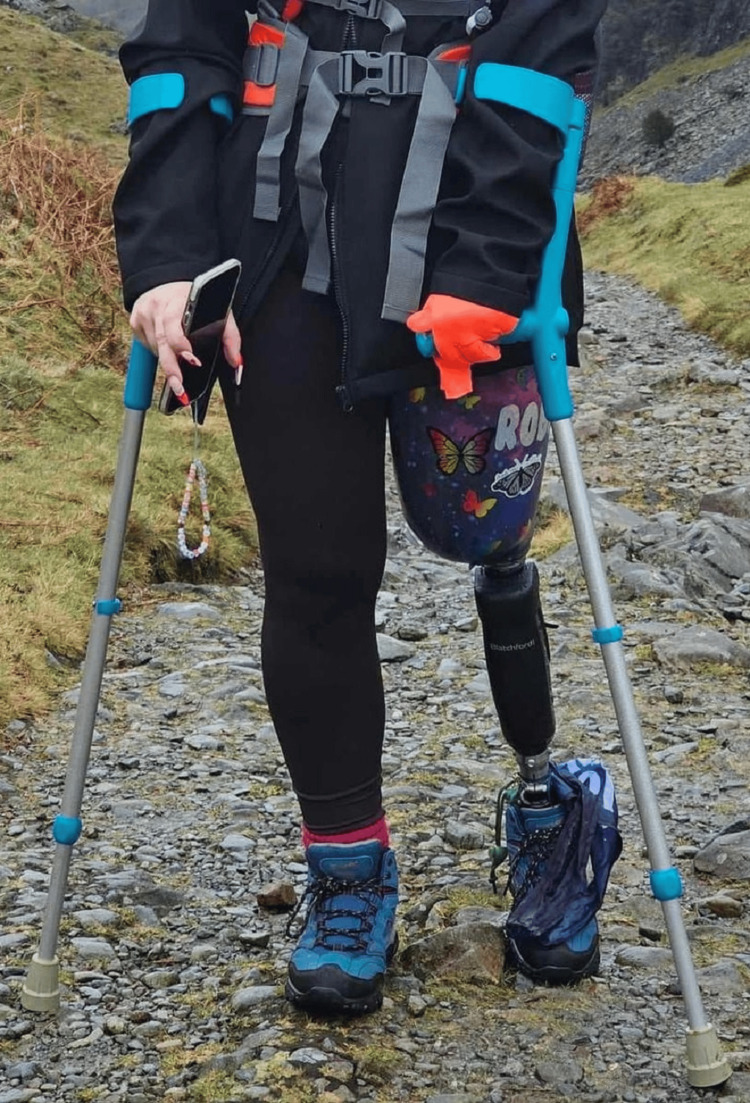
After amputation and fitting of the prosthetic limb Clinical photograph taken six months after the above-knee amputation, demonstrating complete wound healing and good prosthetic adaptation. The patient achieved full functional independence, with a Functional Independence Measure (FIM) score of 120/126, and marked psychological improvement. Her Hospital Anxiety and Depression Scale (HADS) scores improved significantly, with anxiety decreasing from 15 to 6 and depression from 17 to 5, both within normal ranges.

Given the proximal spread of dystonia and the involvement of the knee joint in the abnormal posturing, the surgical team concluded that a below-knee amputation would not adequately address the dystonic pattern or the neuropathic pain distribution, which radiated from the lumbar region downward. After multidisciplinary consultation and detailed preoperative psychological assessment, an above-knee amputation was selected to achieve the best chance of definitive pain relief and restoration of function. In January 2022, after multidisciplinary consultation, she underwent an above-knee amputation of the left limb. Postoperatively, she reported immediate and complete relief of her preoperative pain. She experienced brief daily episodes of phantom limb pain, well-controlled with pregabalin. Following amputation, Figure 4 illustrates the patient’s postoperative status with the fitted microprocessor-controlled prosthetic limb, demonstrating improved limb alignment and function.

She was fitted with a microprocessor-controlled knee prosthesis (MPK) in March 2022 and regained full independence, resuming activities such as running and long-distance walking. Her mental health and self-confidence improved markedly.

At six-month follow-up, the patient had achieved full wound healing and excellent prosthetic adaptation (Figure 4). She was independently ambulant using a microprocessor-controlled prosthesis, capable of walking long distances and performing daily activities without assistance. Her Functional Independence Measure (FIM) score was 120/126, indicating near-complete independence. Psychologically, she demonstrated marked improvement, with HADS scores improving from 15 to 6 for anxiety and from 17 to 5 for depression, both within normal range. She expressed satisfaction with the outcome and reported significant improvement in quality of life, self-confidence, and social engagement.

## Discussion

FDL presents significant diagnostic and therapeutic challenges due to its poorly understood pathophysiology and overlap with other functional neurological disorders. Traditionally, FDL has been classified as a functional movement disorder, which implies the absence of identifiable structural abnormalities of the nervous system. However, emerging evidence suggests that patients with FDL may exhibit functional alterations in central nervous system activity, such as disrupted sensorimotor integration and abnormal body representation, without any structural brain lesions [1,3,5]. These findings do not change the functional classification of the disorder but offer valuable insight into potential underlying mechanisms.

A subset of patients with FDL exhibit persistent desires for limb amputation, a phenomenon described within the framework of body integrity identity disorder (BIID). These individuals may perceive a mismatch between their physical form and internal body image, leading to psychological distress and, in rare cases, self-harm or amputation requests [2,6].

Conservative management, including physical therapy, medication, nerve blocks, and psychological support, often yields limited success. In a landmark study of 103 patients, most showed poor response to traditional therapies, highlighting the chronicity of symptoms [1]. Despite ethical concerns and poor reported outcomes, some patients undergo amputation. 

While amputation has been explored in chronic pain conditions such as CRPS, applying such data to FDL can be misleading due to fundamental differences in pathophysiology, clinical course, and treatment response. In our case, the decision for amputation was based not on extrapolation from CRPS literature, but on the patient's unique clinical presentation of fixed dystonia with persistent, refractory neuropathic pain and severe psychological distress. Previous case reports on limb amputation in FDL - although few - have described mixed outcomes, often contextualized within the framework of BIID [2,6]. Therefore, our discussion of amputation in FDL remains grounded in the limited literature specific to FDL itself, recognizing that conclusions must be cautious due to the rarity of such interventions and the ethical complexity they entail.

Nonetheless, rare cases show that when amputation is combined with thorough psychological screening and multidisciplinary care, it may provide functional and emotional relief. This case should not be interpreted as evidence supporting the general efficacy of amputation for fixed dystonia. Rather, it highlights an exceptional instance where a multidisciplinary team, after exhausting conservative options and carefully assessing psychological readiness, considered amputation for a highly refractory case. Such individualized decisions must remain rare, ethically scrutinized, and supported by careful follow-up. More rigorous research, such as cohort studies or controlled trials - although difficult in rare disorders like FDL - is needed before any recommendations regarding amputation can be made.

Deep brain stimulation and other neuromodulatory interventions have also been explored in extreme cases of chronic pain, although such approaches remain experimental [7]. Additionally, improved diagnostic criteria and pathophysiological insights into CRPS and FDL are contributing to better treatment planning [8].

Phantom limb pain, though common, was mild and controlled, consistent with findings that preoperative mental health and clear expectations influence phantom pain severity [9]. Additionally, advanced prosthetics like microprocessor-controlled knees have been shown to significantly improve gait and confidence in motivated users.

Key takeaways

From a clinical perspective, FDL should be diagnosed only after thorough exclusion of structural, inflammatory, and psychogenic causes. Optimal management requires a stepwise, multidisciplinary approach that integrates pharmacologic therapy, physiotherapy, and psychological support. Surgical interventions, including amputation, should be reserved for exceptional cases where functional recovery and quality-of-life benefits clearly outweigh ethical and physical risks. Ultimately, the management of patients with fixed dystonia requires compassion, coordinated teamwork, and careful ethical consideration to ensure that all therapeutic decisions remain patient-centered and evidence-informed.

## Conclusions

In conclusion, FDL is a rare condition with uncertain pathophysiology and variable treatment outcomes. While this case demonstrated marked subjective improvement after amputation, the findings represent an isolated observation rather than evidence of treatment efficacy. Amputation should remain a last-resort measure, undertaken only after comprehensive multidisciplinary evaluation and failure of all conservative approaches. Further prospective and collaborative research is essential to clarify prognostic factors, guide clinical decision-making, and establish standardized management strategies for this complex disorder.

## References

[1] Schrag A, Trimble M, Quinn N, Bhatia K (2004). The syndrome of fixed dystonia: an evaluation of 103 patients. Brain.

[2] Edwards MJ, Alonso-Canovas A, Schrag A, Bloem BR, Thompson PD, Bhatia K (2011). Limb amputations in fixed dystonia: a form of body integrity identity disorder?. Mov Disord.

[3] Katschnig P, Edwards MJ, Schwingenschuh P, Aguirregomozcorta M, Kägi G, Rothwell JC, Bhatia KP (2010). Mental rotation of body parts and sensory temporal discrimination in fixed dystonia. Mov Disord.

[4] Espay AJ, Lang AE (2015). Phenotype-specific diagnosis of functional (psychogenic) movement disorders. Curr Neurol Neurosci Rep.

[5] van der Laan L, ter Laak HJ, Gabreëls-Festen A, Gabreëls F, Goris RJ (1998). Complex regional pain syndrome type I (RSD): pathology of skeletal muscle and peripheral nerve. Neurology.

[6] First MB (2005). Desire for amputation of a limb: paraphilia, psychosis, or a new type of identity disorder. Psychol Med.

[7] Dielissen PW, Claassen AT, Veldman PH, Goris RJ (1995). Amputation for reflex sympathetic dystrophy. J Bone Joint Surg Br.

[8] Zoing MC, Burke D, Pamphlett R, Kiernan MC (2006). Riluzole therapy for motor neurone disease: an early Australian experience (1996-2002). J Clin Neurosci.

[9] Whyte AS, Niven CA (2001). Psychological distress in amputees with phantom limb pain. J Pain Symptom Manage.

